# Effects of Physical Rehabilitation on Spatiotemporal Gait Parameters and Ground Reaction Forces of Patients with Intermittent Claudication

**DOI:** 10.3390/jcm9092826

**Published:** 2020-08-31

**Authors:** Wioletta Dziubek, Małgorzata Stefańska, Katarzyna Bulińska, Katarzyna Barska, Rafał Paszkowski, Katarzyna Kropielnicka, Ryszard Jasiński, Anna Rachwalik, Marek Woźniewski, Andrzej Szuba

**Affiliations:** 1 Department of Physiotherapy, University School of Physical Education, 35 Paderewskiego Street, 51-612 Wrocław, Poland; wioletta.dziubek@awf.wroc.pl (W.D.); katarzyna.bulinska@awf.wroc.pl (K.B.); ryszard.jasinski@awf.wroc.pl (R.J.); marek.wozniewski@awf.wroc.pl (M.W.); 2 Department of Cardiology, Jelenia Góra Valley Provincial Hospital Center, Ogińskiego 6, 58-501 Jelenia Góra, Poland; katarzyna.zywar@gmail.com; 3 Department of Angiology, Diabetology and Hypertension, Wroclaw Medical University, Borowska 213, 50-556 Wrocław, Poland; rafal.paszkowski@umed.wroc.pl (R.P.); anna.rachwalik@umed.wroc.pl (A.R.); andrzej.szuba@umed.wroc.pl (A.S.); 4 WROVASC—An Integrated Cardiovascular Centre, Specialist District Hospital in Wroclaw, Centre for Research and Development, H. Kamieńskiego 73a, 51-124 Wroclaw, Poland; kropielnicka.k@gmail.com

**Keywords:** peripheral arterial disease, gait analysis, Nordic walking, resistance training

## Abstract

Chronic ischemia of the lower extremities often presents as intermittent claudication characterized by lower limb pain which subsides after a short break. This study aimed to provide an assessment of the spatiotemporal parameters of gait and ground reaction forces in patients with PAD participating in three forms of supervised physical training. A total of 80 subjects completed a three-month supervised physical rehabilitation program with three sessions per week. The subjects were assigned to one of three programs: group 1—standard walking training on a treadmill (TT); group 2—Nordic walking (NW) training; group 3—strength and endurance training comprised of NW with isokinetic resistance training (NW + ISO). Gait biomechanics tests (kinematic and kinetic parameters of gait) and a six-minute walk test were carried out before and after three months of physical training. Nordic walking training led to the greatest improvements in the gait pattern of patients with PAD and a significant increase in the absolute claudication distance and total gait distance. Combined training (NW + ISO) by strengthening the muscles of the lower extremities increased the amplitude of the general center of gravity oscillation to the greatest extent. Treadmill training had little effect on the gait pattern. Nordic walking training should be included in the rehabilitation of patients with PAD as a form of gait training, which can be conducted under supervised or unsupervised conditions.

## 1. Introduction

Atherosclerosis is the most common cause of peripheral arterial disease (PAD) of the lower extremities, resulting in chronic leg ischemia. Chronic ischemia of the lower extremities often presents as intermittent claudication of the calf or the whole leg, characterized by pain after walking a certain distance which subsides after a short break [1,2].

Prolonged ischemia and recurring pain cause a significant reduction in muscle strength, which eventually leads to decreased muscle mass and metabolic changes in their structure [3] Histopathology studies have demonstrated a reduction in the number of fast-twitch fibres and motor units in patients with PAD, which decreases muscle strength and endurance. Furthermore, the gait of patients with PAD is less ergonomic than healthy controls, as it is slower and heavier [4,5].

Pain associated with PAD reduces physical activity, resulting in slower movement, a reduced range of motion in the joints of the lower extremities, and a shorter stride length, which increases the risk of falling [6,7]. In PAD patients, the involvement of particular gait phases changes as the swing phase is reduced and stance phase is prolonged [8], which is probably due to weakness in the ankle and knee muscles [4,9,10].

The treatment of PAD includes the modification of risk factors, pharmacological and surgical interventions, and physical rehabilitation. Lifestyle changes are the primary way to prevent PAD, including stopping smoking, consuming a balanced diet, increasing daily physical activity, and weight reduction [11].

The primary aims of physical rehabilitation are to extend the walking distance until claudication onset and to reduce pain, which can be achieved through regular physical training [12]. Treadmill training is the current gold standard for rehabilitation; however, researchers have observed a non-physiological gait pattern in people with PAD due to their fear of falling [13,14,15]. The most important element of physical rehabilitation is walking training. Therefore, training programs employing forms of supervised walking other than treadmill, in addition to extending the distance until claudication, could also improve the gait pattern of these individuals. Research by Schieber et al. [16] confirmed that a supervised standard walking training program improved the gait biomechanics of PAD patients. However, there is limited information about the forms of physical training that produce results and improve the gait pattern of PAD patients.

Regular physical exercise improves physical fitness, daily functioning, and quality of life in patients with PAD [17,18].

### Study Aim

The aim of this study was to assess the spatiotemporal parameters of gait and ground reaction forces in patients with PAD participating in three different forms of supervised physical training.

## 2. Materials and Methods

This work is part of the “WROVASC—Integrated Center for Cardiovascular Medicine” project, co-financed by the European Regional Development Fund under the Innovative Economy Operational Program 2007–2013, and implemented at the Research and Development Center of the Provincial Specialist Hospital in Wroclaw, Poland.

All subjects gave their informed consent for inclusion before they participated in the study. The study was conducted in accordance with the Declaration of Helsinki, and the protocol was approved by the Ethics Committee of the Medical University of Wroclaw, Poland (reference no. KB-130/2008).

### Sample Characteristics

Recruitment for the rehabilitation program was carried out at healthcare facilities in Wroclaw, Poland. The criteria for inclusion in the rehabilitation program were as follows: aged above 40 years, chronic lower limb ischemia (PAD IIa and IIb according to Fontaine’s classification), claudication distance stable for at least the last 3 months, ankle-brachial index (ABI) lower than 0.9, and written consent to participate in the program.

The exclusion criteria for the rehabilitation program were PAD classifications of Fontaine I (distance without pain, no impairment in walking ability) and III/IV (resting pain/trophic ulcers), uncontrolled hypertension and/or diabetes, acute and chronic decompensated heart failure, cardiovascular event in the year preceding the rehabilitation program, revascularisation procedure in the past 3 months, overall poor health, inability to perform functional motor tests, mental illness, participation in another research program, or participation in less than 70% of the physical training sessions [19]. Patients with degenerative spine/joint disease were excluded if they could not walk 500 m without pain or they had history of spine or joint surgery. The eligibility of patients was decided by a team of angiologists, cardiologists, and physiotherapists.

**Figure 1:**
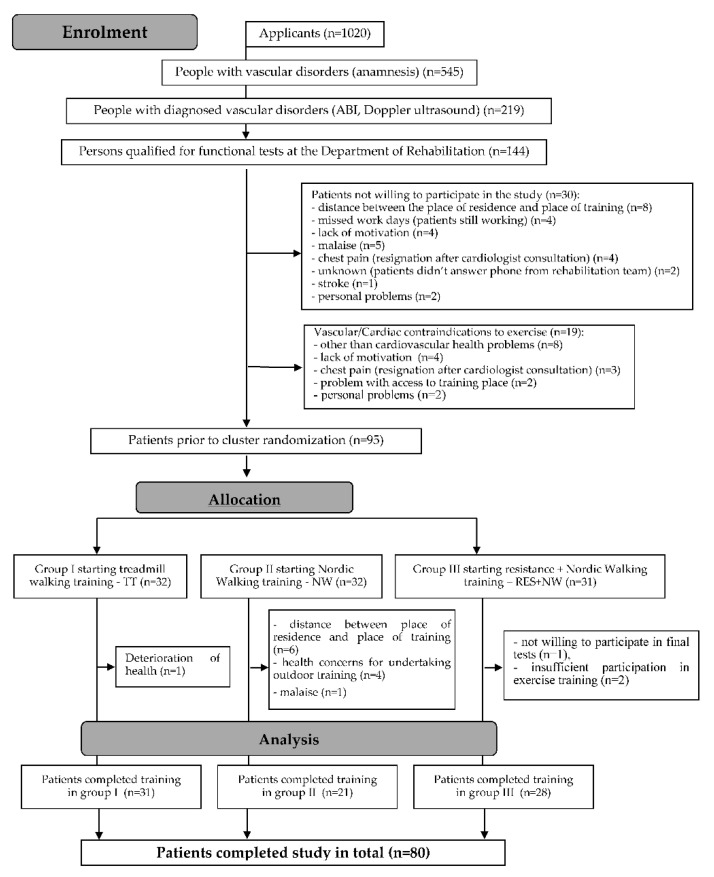
A detailed process of patient recruitment for the rehabilitation program—with the consent of the authors of Kropielnicka et al. [20].

Screening tests were performed on more than 1000 people, of whom 545 were found to have vascular disorders in the lower limbs. Clinical trials were conducted involving 219 people. Before entering the rehabilitation program, detailed angiological examination and risk assessment were carried out for each patient. Those patients with no medical contraindications to perform physical activity were referred for functional tests at the University of Physical Education in Wroclaw, where physical training was conducted for eligible subjects. After consultation with rehabilitation specialists and setting goals with the patient, a 3-month supervised motor rehabilitation program would begin, with patients randomly assigned to one of the three models described below. Medical examinations were carried out on 144 people, out of which 19 did not qualify for functional examinations due to health reasons. Of the 125 individuals who qualified, 30 did not give consent to participate in the rehabilitation program (Figure 1).

A total of 95 subjects participated in a 3-month supervised physical rehabilitation program with three sessions per week. The subjects were assigned to one of three rehabilitation programs using the pseudorandomisation method (consecutive patients were assigned to one group or the other: group 1, standard walking training on a treadmill (TT); group 2, Nordic walking (NW) training; group 3, strength and endurance training comprised of NW combined with isokinetic strength training (NW + ISO). Patients declared no additional systematic physical activity during the training program.

Gait biomechanics tests were carried out at two time points: test 1 was conducted before starting physical training; and test 2 was performed after 3 months of physical training. Both tests were completed by 80 subjects.

The characteristics of patients who qualified for the rehabilitation program are presented in Table 1 and Table 2.

## 3. Study Methods

### 3.1. Gait Tests

#### 3.1.1. Gait Biomechanics Tests

The tests and analyses of gait biomechanics were carried out at the Biomechanics Laboratory in Wroclaw. In order to record kinematic and kinetic parameters of gait, an optoelectronic BTS Smart-E system for three-plane motion analysis (BTS Bioengineering Corp., Quincy, MA, USA) was used, equipped with six digital cameras operating in the infrared range (1.1 μm) with 200-Hz frequency and two AXIS 210A network cameras working in a visible light range with a frequency of 20 Hz. The set up was supplemented with two dynamometric Kistler 9286 platforms (Kistler Group, Winterthur, Switzerland) with 200-Hz signal recording, constituting a central part of the 6-m track. All devices provided measurements synchronously.

Prior to each test, 22 passive photo-reflective markers were attached to the patient’s body, corresponding to selected anthropometric points in accordance with the Davis protocol. Patients performed 10–15 registered walks. Only measurements in which the entire plantar part of each foot was in contact with dynamometer platform during the walk were included in further analysis. Recording was carried out to obtain a minimum of three valid measurements of the ground reaction forces of both feet [19].

A digitally processed image from the cameras enabled the measurement of mean gait velocity (velMEAN [m/s]), number of steps per minute (CADENCE [step/min]), stride length and stride width (length STRIDE [m] and width STRIDE [m]), stride and swing velocity (velSTRIDE [m/s] and velSWING [m/s]), stride time for the swing and stance phases (tSTRIDE [s], tSWING [s] and tSTANCE [s]) and double stride time (tdoubleSTRIDE R [s]). A three-plane measurement of ground reaction forces was used to analyse vertical loading during initial contact (VEmax1), loading response (VEmin) and toe-off (VEmax2), in addition to the anteroposterior (AP) load distribution (APmax and APmin) and mediolateral (ML) load distribution (MLmax and MLmin). For further analysis, ground force responses were also presented as a percentage of body weight [% BW].

#### 3.1.2. Six-Minute Walk Test (6MWT)

A six-minute walk test was conducted on a marked 30-m corridor. The test consisted of an easy walk at a pace that the patient uses daily. In the case of people with intermittent claudication in whom the occurrence of maximal pain forced them to stop during the test, measured time was not stopped.

Before the test, respondents were informed of the possibility of resting during the test in a sitting or standing position (on chairs located respectively at distances of 1, 15, and 30 m) when symptoms of exercise intolerance intensified (dyspnea, shortness of breath, maximal pain of the lower limbs, general fatigue, etc.). The entire test procedure was carried out by an experienced physiotherapist with medical supervision.

If, during the test, subjects experienced extremely severe symptoms of exercise intolerance, which, despite short rest did not subside, the test was discontinued immediately.

Test results consisted of a measured in meters (with 1 m accuracy) claudication and total distance.

### 3.2. Physical Rehabilitation of Patients

Physical rehabilitation was carried out for a period of 12 weeks, with three sessions per week (36 sessions total) of 45 min duration, with additional time to perform measurements of hemodynamic parameters (blood pressure and heart rate). The three types of training administered to groups 1, 2, and 3 are described.

Standard (TT) treadmill training (group TT) was carried out in accordance with the TASC II recommendations. The patient walked on a treadmill (HX-100 model) at a constant speed of 3.2 km/h at a constant slope of 12%. The workout was based on interval training. The walking time was determined by the occurrence of submaximal claudication pain (i.e., level 4 according to the 5-grade scale of the American Collage of Sport Medicine), at which point the patient stepped onto the motion-free side strip of the treadmill and rested until the pain subsided. However, the rest time did not exceed 2 min. As the patient’s walking functions improved, the training was extended so that the patient could walk the longest distance possible in 45 min. The training was individualized.

Nordic walking training (group NW) was run by experienced NW instructors in accordance with the International Nordic Walking Association (INWA) guidelines. At the beginning of training, a general warm-up (up to 10 min) was performed using NW poles (KV + Campra Clip), followed by NW training using the interval method based on the same principles as those used for treadmill training. At the end of the training unit, stretching and breathing exercises were performed (5 min). The training took place in groups of up to 12 people.

Combined training (group ISO + NW) consisted of isokinetic training performed and NW (ISO + NW) alternately (e.g., Monday, NW; Wednesday, ISO; Friday, NW; Monday, ISO, etc.). NW training was conducted according to the same principles described above for group 2. Isokinetic (resistance) training was conducted under constant angular velocity of movement, ensuring constant resistance, with biofeedback data provided by the Biodex S4 Pro device (Biodex Medical Systems, Inc., USA). Before commencing the actual training, the patient performed five alternating, maximum flexion and extension movements of the ankle and knee joints with each resistance placed. The training load was assumed at 70–80% of the maximum value (Nm) for both muscle groups (plantar and dorsal flexors of the ankle; training for flexors and extensors of both knee joints was introduced for patients with considerable arterial closure). The use of biofeedback during training allowed movements with the strength required to reach the set targets.

The following velocities were used: 60, 120, 180, 240, 300, and 300, 240, 180, 120, 60°/s (in line with the pyramid rules). For each resistance, the patient performed 10 alternating flexion and extension movements of these joints, totalling 100 movements per extremity. After each velocity, the patient rested their foot or knee in the intermediate position for 1 min. The time was extended in the event that claudication pain took longer to subside.

The NW training took place in a group, whereas the isokinetic training was performed individually at the Functional Research Laboratory of the Faculty of Physiotherapy of the University of Physical Education in Wroclaw.

### 3.3. Statistical Analysis

The distribution of all examined parameters was verified using the Shapiro–Wilk test, which confirmed close to normal distribution in almost each of the studied groups. The Levene’s test confirmed the homogeneity of variance. Descriptive statistics and mean percentage differences in values obtained before and after the training intervention were calculated. The homogeneity of the study groups was verified using the Chi^2^ Test. In each of the studied groups, the mean differences between values recorded in test 1 (before training) and test 2 (after the training cycle) were statistically verified using the T-test or U Mann–Whitney Test. In order to compare the rehabilitation methods, repeated-measures analysis of variance (ANOVA) was used.

## 4. Results

Baseline analysis did not show any statistically significant differences between the subject’s gait values for both right and left extremities. There were also no statistically significant differences in gait parameters between sides with a lower or higher ABI. Therefore, further analyses were conducted without taking into account the dominant limb and without specifying the side with a higher or lower ABI.

In order to evaluate the efficacy of the rehabilitation methods, the mean values and confidence interval between spatiotemporal parameters describing the subjects’ gait before and after therapy were calculated. The greatest differences were observed in the NW group for both right and left limbs (Table 3). The greatest increases occurred in gait velocity parameters (mean velocity, velMEAN; stride velocity, velSTRIDE; and swing velocity, velSWING) and stride length (lSTRIDE). Large reductions in stride time (tSTRIDE) and swing time (tSWING) were also observed.

In each of the studied groups, significant differences in the mean spatiotemporal gait parameters were observed before and after the three-month training period. In each of the groups, gait velocity and stride length increased, while the duration of individual phases shortened. The most statistically significant changes were observed in the NW group (Table 4). However, no statistically significant differences were found between the groups undertaking different training models, and there was also no significant interaction between the type of training and test number (pre- and post-intervention; Table 5).

The three-plane measurement of ground reaction forces during walking was also performed twice. For both the right and left limbs, the largest change between the first and second tests was observed in the NW group, especially for values recorded in the AP plane (Table 6 and Table 7).

The ANOVA confirmed that the differences in parameters recorded before and after therapy were statistically significant. However, the type of training and the interaction between training and test number did not reach statistical significance (Table 8).

There was observed a statistically significant increase in total distance in the 6-Minute Walk Test in each test group after training: by 13% in the ISO + NW group, 11% in the NW group and 9% in the TT group. The total distance of intermittent claudication increased also in all groups. The registered difference turned out to be significant only in the group participating in the Nordic walking training (Table 9).

## 5. Discussion

Gait is a basic human motor function that plays an important role in everyday life. Chronic lower limb arterial insufficiency impairs the walking capabilities of affected individuals, which has a negative impact on their quality of life [21]. PAD is frequently associated with pain and discomfort, speed limitations and shortening of stride length, thereby reducing the distance covered [22,23,24,25].

In addition to reducing the strength of the lower limb muscles [10,11,12,13,14,15,16,17,18,19,20,21,22,23,24,25,26], chronic lower limb ischemia also changes the gait pattern. Compared to a group of healthy individuals, people with PAD exhibit an extended stance phase while simultaneously shortening the swing phase [8]. These abnormalities occur both during pain-free gait and after the onset of claudication symptoms [9,27]. Angular changes in the lower limb joints of PAD patients while walking point to disorders of the neuromuscular system, which limits muscular coordination while increasing the risk of falls [27].

A three-plane study by Scott-Pandorf et al. [28], in which ground reaction forces during gait were analysed in patients with PAD compared to an asymptomatic control group, demonstrated a flattening of the curve representing the vertical force. This is due to a decrease in oscillation of the center of gravity of the body, which explains why gait becomes less stable. In the AP plane, there is a decrease in the toe-off force, an increase in force in the medial direction and a decrease in the lateral direction in the transverse plane [28]. It is presumed that this is due to pain and limited neuromuscular control of the lower limbs, which causes PAD patients to walk on a broader foot base and limits their foot propulsion movement [29]. The observed changes in the gait of PAD patients may also result from a decrease in the strength and endurance of the thigh and shin muscles [9,28] caused by the atrophy of muscle fibres and replacement with connective and adipose tissue [30,31,32]. In addition, it has been shown that claudication pain can recruit more shin muscle motor units in a non-ergonomic way, leading to faster fatigue [33].

As mentioned earlier, gait disturbance is one of the key elements that reduces the quality of life of PAD patients. Therefore, the main goal of the rehabilitation process is to improve gait quality and reduce pain. Gait training on a treadmill is a standard and generally accepted rehabilitation method [34,35]. However, despite achieving beneficial effects, a few authors have highlighted some limitations of the unnatural conditions associated with treadmill training [36,37,38]. It is believed that the fear of falling, stooped posture, shortening of stride, and infrequent use of upper limbs due to gripping the treadmill, as well as the unchanging visual environment despite moving, may adversely affect the walking pattern. Lee and Hindler [38] compared gait on a treadmill and on a regular surface, and revealed that the most changes took place in the sagittal plane joint moments, where apart from the plantar flexion, changes occurred in all joints during particular gait phases, to the detriment of a treadmill. Additionally, in the stance phase, the anterior tibial and gastrocnemius muscles are less active, while in the stance and swing phases, the thigh muscle exhibits greater activity when walking on a treadmill compared to walking on a regular surface [38]. Our research demonstrated that treadmill training (standard) did not significantly improve the gait pattern of participants, which partly confirms our assumptions.

Nordic walking is a more natural form of gait training, which has increasingly been used in the rehabilitation of patients with PAD [17,39,40]. NW is low cost, and training can be conducted under supervised or unsupervised conditions, individually or in groups. Furthermore, it is beneficial due to the changing environment and, according to research [36,41,42], a patient walking at the same intensity does not feel as tired as they do when walking without poles.

We did not identify any clear advantages of one method of PAD rehabilitation over the others. A meta-analysis conducted by Golledge et al. [17] comparing the maximum walking distance achieved during a treadmill test and a six-minute corridor test showed no significant differences between standard and NW training. However, research conducted by Girold et al. [43] and Oakley et al. [40] demonstrated greater efficacy of NW training in extending the walking distance. The results of a study by Collins et al. [39] were slightly different, showing a greater improvement in gait endurance during training without poles compared to NW training. Metabolic changes in muscle fibres resulting from chronic ischemia of the lower limbs leads to a reduction in the strength, endurance and power generated by the muscles [44], thus rehabilitation of PAD patients has been supplemented with resistance training [20,32,45]. The comparison of resistance training with standard treadmill training in a meta-analysis by Parmenter et al. [46] also did not give an unequivocal answer for which rehabilitation method gives better results in extending the claudication distance and total distance in patients with PAD.

A small number of studies on the changes in gait pattern as a result of rehabilitation of PAD patients [15,16,47,48] prompted us to evaluate the effectiveness of three rehabilitation programs: standard treadmill training (S), Nordic walking (NW), and training that combines NW with resistance training under isokinetic conditions (NW + ISO).

In this research, three-dimensional gait analysis revealed the greatest improvement in the group that undertook NW training and, to a slightly lesser extent, in the group that participated in combined training. Gait velocity increased in both groups, and most of the velocity and time parameters describing the individual phases of gait were improved. Additionally, in the NW group, we observed a significant increase in stride. The described changes indicate an improvement in the gait pattern of patients with PAD. This is probably due to differences in NW training volume between the two groups. In the ISO +NW group the whole training volume was divided between NW (50%) and ISO (50%) time, as opposed to the NW group (100% training time). NW training involves the same muscle groups used for gait, hence the resulting significantly greater improvement in this group of gait parameters. However, at the beginning of learning NW technique, the practitioner must be mindful that this is an unfamiliar form of movement. One of the most important elements of the technique is the elongation of stride and active loading response of the foot, from the heel to the toes, as well as the moment of toe-off which gives the body momentum (learning technique available at https://www.inwa-nordicwalking.com/). Therefore, the acquired NW gait technique led to improvements in spatiotemporal parameters in particular phases of gait, similar to isokinetic training, which aimed to strengthen the muscles of the dorsal and plantar flexors of the foot. A lack of research on gait pattern among PAD patients undertaking NW with combined training (NW alternately with isokinetic training) does not allow for verification of the above results. However, based on an analysis of spatiotemporal parameters, NW lengthens the stride, shortens the stride time, improves gait regularity, and decreases the frequency of steps of healthy participants [49,50]. Additionally, research by Kocur et al. [48] confirmed beneficial effects on postural control, which may help to reduce the risk of falls. Furthermore, strengthening the muscles of the ankle joint has a direct impact on extending the maximum walking distance in people with intermittent claudication, which was confirmed in a study by Kropielnicka et al. [20].

In our study, all three types of training extended total walking distance reached in a six-minute walk test, but only Nordic walking training had an influence on significant changes in claudication distance. In some studies, there is evidence that Nordic walking training brings better outcomes in claudication distance than treadmill training [20,37,41]. Interestingly, there is no statistical confirmation for claudication distance after combined training (Nordic walking with resistance training). Parmenter et al. [46], comparing resistance training and treadmill training, also confirmed no statistical changes in this parameter. Authors suggest that Nordic Walking trains whole body not only isolated parts (lower limbs), so there is bigger influence on exercise capacity and maximal oxygen consumption [39,50]. It probably has an impact on lowering the pain threshold during walking [51]. Analysis of ground reaction forces confirmed that the NW group achieved an increase in AP strength during both initial contact and toe-off phases, which is probably due to the strengthening of shin muscles and the increase in gait pace. Additionally, a reduction in vertical force in the middle of the stance phase (VEmin) was observed in the combined training group, which indicates an increase in the vertical oscillation of the center of gravity. This is probably the result of increased muscle strength of the lower limbs, which affects the dynamic stability during gait [28]. When comparing ordinary walking with NW. Park et al. [49] also demonstrated a decrease in vertical force in the middle of the stance phase during NW, along with increases during the initial and final stance phases. The authors attributed these changes to the increase in gait pace associated with the NW technique and to the strengthening of shin muscles [49] which supports the changes in ground reaction forces in the AP plane observed in the current study.

The ANOVA did not reveal any significant differences between the groups; however, intragroup analysis of the spatiotemporal parameters of gait revealed the most significant improvements in groups participating in NW training. The standard treadmill training, despite the obvious health benefits of the exercise itself, did not have a considerable impact on the walking pattern of participants. The improvements observed in this group were only related to time parameters for the left limb and the vertical ground reaction force in the final stance phase for the right limb. The meaning of these findings is not entirely clear, and this should be investigated in further studies.

In response to a six-month treadmill workout program, Schieber et al. [16] showed an improvement in the medial-lateral ground reaction force upon initial heel contact, which could be confirmed by increases in hip extensor torque and in power absorption generated by the ankle joint. During the foot propulsion movement, power increased in the ankle and hip joints [16]. However, it should be mentioned that these researchers used a protocol other than TASC II, which is recommended for the rehabilitation of patients with PAD, and another measurement system for gait analysis. An improvement in stride length, shorter stride duration in both lower limbs and a decrease in the frequency of steps was observed by Konik et al. [15] following a three-month treadmill training program. In contrast, no changes in kinetic and kinematic parameters of gait were observed in a study by King et al. [47] following a three-month rehabilitation program consisting of walking training supplemented with aerobic and strength exercises. Considering the results of this research, it can be concluded that training based on a correct NW technique improves the walking pattern of PAD patients. Training on a treadmill did not bring significant results. Therefore, NW training should be considered in the rehabilitation program of patients with PAD, especially as this particular form of movement can be performed without supervision at home. The main goal in clinical practice is to improve both walking distance and gait disturbances.

## 6. Conclusions

1. Nordic walking training led to greater improvements in the gait pattern of patients with PAD compared to combined and standard training.
2. Combined training (NW + ISO) corrected the walking pattern to a lesser extent than NW training. However, by strengthening the muscles of the lower extremities, it increased the amplitude of the general center of gravity oscillation to the greatest extent.
3. Treadmill training had little effect on the gait pattern of PAD patients.
4. Nordic walking training should be included in the rehabilitation of patients with PAD as a form of gait training, which can be conducted under supervised or unsupervised conditions.

## Figures and Tables

**Table 1:** Patient characteristics.

	Group TT (*n* = 31)		Group NW (*n* = 21)		Group ISO + NW (*n* = 28)		*p*
	Average	SD	Average	SD	Average	SD	
Age [years]	67.00	7.43	67.00	9.32	67.82	8.49	0.92
Height [cm]	168.03	8.56	166.81	7.40	169.54	9.07	0.53
Body weight [kg]	79.02	14.31	74.44	12.44	78.84	15.43	0.47
ABI R	0.68	0.19	0.76	0.17	0.76	0.19	0.14
ABI L	0.68	0.16	0.71	0.22	0.70	0.19	0.84

ABI—ankle brachial index; R—right lower limb, L—left lower limb; statistically significant value (*p* < 0.05).

**Table 2:** Comorbidities and PAD levels of occlusion.

	Group TT (*n* = 31)	Group NW (*n* = 21)	Group ISO + NW (*n* = 28)	Chi^2^ Test *p*
Smoking:				
smokers	9(29%)	9(42.9%)	7(25%)	0.5030
active/past smokers	6/3	5/4	5/2	
Diabetes type 2	12(38.7%)	8(38.1%)	12(42.9%)	0.5630
Hypertension	26(83.9%)	14(66.7%)	20(71.4%)	0.0016 *
Revascularisation	11(35.5%)	7(33.3%)	8(28.6%)	0.8442
Degenerative changes of the spine and/or peripheral joints	12(38.7%)	4(19.0%)	8(28.6%)	0.2180
PAD type (*n*)	26 FP (83.9%) 4 AI (12.9%) 1 ML (3.2%)	16 FP (76.2%) 3 AI (14.3%) 1 ML (4.89%) 1 P (4.8%)	23 FP (82.1%) 2 AI (7,1%) 1 ML (3.6%) 2 P (7.1%)	

FP–femoral-popliteal, AI–aortoiliac, ML-multi-level; P–peripheral; * statistically significant value (*p* < 0.05).

**Table 3:** The values of the spatiotemporal parameters of gait recorded in each group of subjects before and after 3-month training program.

	Group TT				Group NW				Group ISO + NW			
	Before		After		Before		AFter		Before		After	
	Mean	±SD	Mean	±SD	Mean	±SD	Mean	±SD	Mean	±SD	Mean	±SD
velMEAN [m/s]	0.92	0.14	0.95	0.13	0.87	0.11	0.97	0.16	0.94	0.16	0.99	0.18
CADENCE (step/min)	99.22	10.84	101.09	9.16	101.22	11.08	104.09	8.81	100.66	10.81	99.88	8.45
velSTRIDE R [m/s]	0.93	0.14	0.96	0.13	0.88	0.13	0.98	0.16	0.96	0.16	1.01	0.18
velSTRIDE L [m/s]	0.93	0.14	0.96	0.13	0.88	0.12	0.98	0.16	0.96	0.16	1.01	0.18
velSWING R [m/s]	2.30	0.29	2.36	0.29	2.23	0.27	2.49	0.32	2.31	0.40	2.5	0.37
velSWING L [m/s]	2.28	0.30	2.35	0.28	2.18	0.26	2.46	0.31	2.34	0.41	2.51	0.40
tSTRIDE R [s]	1.20	0.11	1.18	0.11	1.20	0.12	1.15	0.10	1.24	0.12	1.19	0.12
tSTRIDE L [s]	1.20	0.11	1.19	0.11	1.20	0.11	1.15	0.10	1.24	0.12	1.18	0.12
tSWING R [s]	0.43	0.05	0.42	0.04	0.42	0.06	0.40	0.04	0.43	0.03	0.42	0.04
tSWING L [s]	0.43	0.04	0.42	0.04	0.42	0.05	0.40	0.04	0.42	0.03	0.41	0.04
tSTANCE R [s]	0.77	0.08	0.76	0.08	0.77	0.06	0.76	0.07	0.77	0.08	0.79	0.09
tSTANCE L [s]	0.78	0.08	0.76	0.09	0.78	0.09	0.76	0.08	0.77	0.09	0.79	0.09
tdoubleSTRIDE R [s]	1.20	0.11	1.18	0.12	1.19	0.12	1.16	0.10	1.20	0.13	1.21	0.11
tdoubleSTRIDE L [s]	1.20	0.11	1.18	0.12	1.19	0.12	1.16	0.10	1.21	0.13	1.21	0.11
lenght STRIDE R [m]	0.50	0.05	0.51	0.05	0.47	0.06	0.50	0.08	0.53	0.05	0.51	0.08
lenght STRIDE L [m]	0.51	0.05	0.52	0.06	0.47	0.06	0.51	0.07	0.53	0.06	0.52	0.07
widht STRIDE R [m]	0.16	0.02	0.16	0.02	0.15	0.02	0.15	0.02	0.16	0.02	0.16	0.02
widht STRIDE L [m]	0.16	0.02	0.16	0.02	0.15	0.02	0.15	0.02	0.16	0.02	0.16	0.02

vel—velocity; t—time; R—right; L—left; ±SD%— standard deviation.

**Table 4:** Mean differences in the spatiotemporal parameters of gait for each of the groups between tests 1 and 2.

	TT	NW	ISO + NW
velMEAN [m/s]	0.1043	0.0066 *	0.0237 *
CADENCE (step/min)	0.0718	0.2577	0.4658
velSTRIDE R [m/s]	0.1305	0.0074 *	0.0356 *
velSTRIDE L [m/s]	0.1539	0.0040 *	0.0584
velSWING R [m/s]	0.1676	0.0027 *	0.0133 *
velSWING L [m/s]	0.1495	0.0010 *	0.0295 *
tSTRIDE R [s]	0.1001	0.0272 *	0.0167 *
tSTRIDE L [s]	0.0386 *	0.0366 *	0.0108 *
tSWING R [s]	0.4291	0.0198 *	0.0608
tSWING L [s]	0.4069	0.0150 *	0.2339
tSTANCE R [s]	0.0922	0.1980	0.4125
tSTANCE L [s]	0.0474 *	0.7493	0.3870
tdoubleSTRIDE R [s]	0.0889	0.2687	0.4018
tdoubleSTRIDE L [s]	0.0378 *	0.3148	0.3555
length STRIDE R [m]	0.9314	0.0126 *	0.8037
length STRIDE L [m]	0.6693	0.0041 *	0.7205
width STRIDE R [m]	0.7868	0.7245	0.6898
width STRIDE L [m]	0.7868	0.7245	0.6898

* statistically significant value (*p* < 0.05); vel—velocity; t—time; R—right; L—left.

**Table 5:** Repeated-measures ANOVA for the time parameters of gait, taking into account the type of training and the test number.

	TT	NW	ISO + NW
velMEAN [m/s]	0.1043	0.0066 *	0.0237 *
CADENCE (step/min)	0.0718	0.2577	0.4658
velSTRIDE R [m/s]	0.1305	0.0074 *	0.0356 *
velSTRIDE L [m/s]	0.1539	0.0040 *	0.0584
velSWING R [m/s]	0.1676	0.0027 *	0.0133 *
velSWING L [m/s]	0.1495	0.0010 *	0.0295 *
tSTRIDE R [s]	0.1001	0.0272 *	0.0167 *
tSTRIDE L [s]	0.0386 *	0.0366 *	0.0108 *
tSWING R [s]	0.4291	0.0198 *	0.0608
tSWING L [s]	0.4069	0.0150 *	0.2339
tSTANCE R [s]	0.0922	0.1980	0.4125
tSTANCE L [s]	0.0474 *	0.7493	0.3870
tdoubleSTRIDE R [s]	0.0889	0.2687	0.4018
tdoubleSTRIDE L [s]	0.0378 *	0.3148	0.3555
length STRIDE R [m]	0.9314	0.0126 *	0.8037
length STRIDE L [m]	0.6693	0.0041 *	0.7205
width STRIDE R [m]	0.7868	0.7245	0.6898
width STRIDE L [m]	0.7868	0.7245	0.6898

* statistically significant value (*p* < 0.05); vel—velocity; t—time; R—right; L—left.

**Table 6:** The values of the ground reaction forces recorded in each group of subjects before and after the 3-month training program.

	Group TT				Group NW				Group ISO + NW			
	Before		AFter		Before		After		Before		After	
	Mean	±SD	Mean	±SD	Mean	±SD	Mean	±SD	Mean	±SD	Mean	±SD
VE max1 R [%BW]	98.06	4.30	98.02	4.10	99.19	5.11	100.06	5.30	101.27	5.57	99.79	5.06
VE max2 R [%BW]	104.28	5.82	105.87	4.99	104.43	6.85	104.96	8.80	105.30	4.47	104.32	4.57
VE min R [%BW]	86.65	4.33	85.85	3.93	86.31	4.88	84.22	6.58	89.40	5.32	83.89	5.98
VE max1 L [%BW]	99.22	3.89	99.09	3.78	98.20	4.30	100.19	4.64	99.62	5.43	101.9	5.55
VE max2 L [%BW]	103.97	4.51	104.94	4.70	103.22	5.82	105.01	8.31	104.75	5.07	104.72	5.15
VE min L [%BW]	85.87	4.38	86.23	4.69	87.88	3.75	84.63	6.54	88.38	4.78	82.70	5.36
AP max R [%BW]	13.53	2.42	14.06	2.76	12.70	3.27	14.09	2.71	14.83	3.57	14.88	2.74
AP min R [%BW]	−11.10	2.83	−11.87	2.78	−10.45	2.39	−12.10	2.45	−12.01	3.76	−12.26	3.15
AP max L [%BW]	13.33	2.31	13.75	2.31	12.55	2.56	13.93	3.86	13.65	3.80	14.8	3.01
AP min L [%BW]	−11.56	2.42	−11.99	2.51	−9.71	1.83	−11.56	2.79	−12.11	3.15	−12.71	3.04
ML max R [%BW]	5.77	1.47	5.79	1.58	5.55	1.32	5.68	1.65	6.44	1.38	6.14	1.37
ML min R [%BW]	−2.35	0.98	−2.45	1.09	−2.35	0.95	−2.76	1.39	−2.52	1.20	−2.69	1.19
ML max L [%BW]	5.77	1.47	5.79	1.58	5.55	1.32	5.68	1.65	6.44	1.38	6.14	1.37
ML min L [%BW]	−2.35	0.98	−2.45	1.09	−2.35	0.95	−2.76	1.39	−2.52	1.20	−2.56	1.31

BW—body weight; VE—vertical force; AP—anterior-posterior force; ML—medio-lateral force; R—right; L—left side; ±95%—confidence interval.

**Table 7:** Mean differences in ground reaction forces between tests 1 and 2 for each of the groups.

	TT	NW	ISO + NW
VE max1[%BW]R	0.8324	0.5827	0.3778
VE max2[%BW]R	0.0198 *	0.5844	0.4051
VE min[%BW]R	0.2547	0.2364	0.0030 *
VE max1[%BW]L	0.8488	0.0536	0.2537
VE max2[%BW]L	0.1284	0.1341	0.9672
VE min[%BW]L	0.6223	0.0092 *	0.0035 *
AP max[%BW]R	0.1425	0.0455 *	0.6995
AP min[%BW]R	0.1342	0.0216 *	0.5901
AP max[%BW]L	0.2990	0.0151 *	0.1678
AP min[%BW]L	0.3950	0.0006 *	0.7195
ML max[%BW]R	0.9008	0.7549	0.5326
ML min[%BW]R	0.6001	0.1927	0.8457
ML max[%BW]L	0.9008	0.7549	0.5326
ML min[%BW]L	0.6001	0.1927	0.9201

* statistically significant value (*p* < 0.05); BW—body weight; VE—vertical force; AP—anterior-posterior force; ML—medio-lateral force; R—right; L—left side.

**Table 8:** Repeated-measures ANOVA for the ground reaction forces generated during the stance phase, taking into account the type of training and the test number.

ANOVA.	TRAINING	TEST	INTERACTION (Training&Test)
VE max1 R [%BW]	NS	NS	NS
VE max2 R [%BW]	NS	NS	NS
VE min R [%BW]	NS	0.0003 *	0.0180 *
VE max1 L [%BW]	NS	0.0466 *	NS
VE max2 L [%BW]	NS	NS	NS
VE min L [%BW]	NS	0.0000 *	0.0014 *
AP max R [%BW]	NS	0.0201 *	NS
AP min R [%BW]	NS	0.0132 *	NS
AP max L [%BW]	NS	0.0017 *	NS
AP min L [%BW]	0.0453 *	0.0031 *	0.0178 *
ML max R [%BW]	NS	NS	NS
ML min R [%BW]	NS	NS	NS
ML max L [%BW]	NS	NS	NS
ML min L [%BW]	NS	NS	NS

* statistically significant value (*p* < 0.05), NS—result statistically not significant; VE—vertical force; AP—anterior-posterior force; ML—medio-lateral force; R—right; L—left; BW—body weight.

**Table 9:** Total distance and intermittent claudication (CI) distance values during the 6-Minute Walk Test.

		Before			After			*p*
		Mean	Median	SD	Mean	Median	SD	
TT	Total distance [m]	343.50	358.00	66.46	375.11	380.00	69.79	0.0002 *
	CI distances ** [m]	167.35	155.00	111.23	182.27	200.00	122.82	0.6406
NW	Total distance [m]	354.00	350.00	56.07	392.52	397.00	63.88	0.0011 *
	CI distances ** [m]	149.07	125.00	92.15	181.62	200.00	103.21	0.0290 *
ISO + NW	Total distance [m]	374.18	368.00	69.01	422.77	406.50	64.65	0.0007 *
	CI distances ** [m]	155.82	138.50	114.81	193.68	200.00	125.04	0.3507

* statistically significant value (*p* < 0.05); *p*—coefficient of *t*-test (normal distribution **) or U Mann Whitney Test (not normal distribution).
